# Pre-selection of markers for genomic selection

**DOI:** 10.1186/1753-6561-5-S3-S12

**Published:** 2011-05-27

**Authors:** Torben Schulz-Streeck, Joseph O Ogutu, Hans-Peter Piepho

**Affiliations:** 1 Bioinformatics Unit, Institute of Crop Science, University of Hohenheim, Fruwirthstrasse 23, 70599 Stuttgart, Germany

## Abstract

**Background:**

Accurate prediction of genomic breeding values (GEBVs) requires numerous markers. However, predictive accuracy can be enhanced by excluding markers with no effects or with inconsistent effects among crosses that can adversely affect the prediction of GEBVs.

**Methods:**

We present three different approaches for pre-selecting markers prior to predicting GEBVs using four different BLUP methods, including ridge regression and three spatial models. Performances of the models were evaluated using 5-fold cross-validation.

**Results and conclusions:**

Ridge regression and the spatial models gave essentially similar fits. Pre-selecting markers was evidently beneficial since excluding markers with inconsistent effects among crosses increased the correlation between GEBVs and true breeding values of the non-phenotyped individuals from 0.607 (using all markers) to 0.625 (using pre-selected markers). Moreover, extension of the ridge regression model to allow for heterogeneous variances between the most significant subset and the complementary subset of pre-selected markers increased predictive accuracy (from 0.625 to 0.648) for the simulated dataset for the QTL-MAS 2010 workshop.

## Background

Genomic selection (GS) is a method for predicting breeding values on the basis of a large number of molecular markers [1]. However, if many markers actually have zero effects but are estimated to be non-zero, then their cumulative effects increase noise in the estimates [2]. Thus, markers are most useful for GS if they are in high linkage disequilibrium with a QTL. Many authors pre-screen markers before including them in GS (e.g. [3,4]). If a marker is in high linkage disequilibrium with a QTL its effect should be consistent among crosses (full sib families) or generations. One option therefore is to select against markers with inconsistent effects.

We compare different methods for selecting the most relevant markers for GS. Genomic breeding values (GEBVs) were estimated using different BLUP methods and number of pre-selected markers. Besides ridge regression (RR), spatial models were also used. The best model was selected using cross-validation (CV).

## Methods

### Data

A simulated dataset of 3226 individuals in five generations generated for the QTL-MAS 2010 workshop was analysed. A total of 2326 individuals belonging to the first four generations were phenotyped and genotyped with 10031 SNP markers. Moreover, 900 individuals in the fifth generation were genotyped but had no phenotypic records. We focus here only on the quantitative trait. A SNP was included in the analysis only if its minor allele frequency exceeded 2.5%. This resulted in the exclusion of 461 SNPs.

The marker covariate *z_ik_* for the *i*-th individual (*i* = 1, 2,…, *G*) and the *k*-th marker (*k*= 1, 2,…, *M*) for biallelic SNP markers with alleles *A*_1_ and *A*_2_ was set to 1 for *A*_1_*A*_1_, -1 for *A*_2_*A*_2_ and 0 for *A*_1_*A*_2_. Covariates were stored in a matrix ***Z*** = {*z_ik_*}.

### Pre-selection of SNPs

We tested the effect of each SNP on the quantitative trait using three different methods.

#### Method 1

Each SNP was tested using a linear regression, like in Macciotta et al. [4], given by

*y_i_* = *μ* + *u_k_z_ik_* + *e_i_*,

where *y_i_* is the phenotypic record for the *i*-th individual, *μ* is the intercept, *z_ik_* is the genotype of the *i*-th individual for the *k*-th marker, *u_k_* is the slope of the linear regression on the *k*-th marker and *e_i_* is the residual error .

#### Method 2

Each SNP was analysed for consistency among crosses using the model

*y_ic_* = *μ* + *u_k_z_ik_* + *Cross_c_* + *γ_ck_z_ik_* + *e_ic_*,

where *Cross_c_* is the random effect of the *c*-th cross and *γ_ck_* is the slope of the random linear regression of the *c*-th cross on the *k*-th marker. The variance-covariance structure for the random regression was assumed to be unstructured and bivariate-normal (BVN), i.e. , where *Σ* is an unstructured 2×2 variance-covariance matrix. The random interaction effect (*γ_ck_*) served as the error term for the test of the SNP main effect (*u_k_*). If the SNP main effect is highly consistent, the interaction will be small, and so the F-value will be relatively large. Conversely, if the SNP is inconsistent, the main effect will be small and the interaction large, yielding small F-values.

#### Method 3

Each SNP was analysed for consistency among generations using the model

*y_ig_* = *μ* + *u_k_z_ik_* + *Generation_g_* + *γ_gk_z_ik_* + *e_ig_*,

where *Generation_g_* is the random effect of the *g*-th generation and *γ_gk_* is the slope of the random linear regression of the *g*-th generation on the *k*-th marker. Similarly to method 2, the SNP main effect (*u_k_*) is tested against the random interaction term (*γ_gk_*).

The *n* (*n* = 500, 1000, 2000, 3000) most significant markers (i.e. those with the smallest p-values) were included in the GS model.

### GEBVs estimation

The genotypic effect was estimated using the following linear mixed model:

*y_i_* = *μ* + *g_i_* + *e_i_*,

where *y_i_* is the phenotypic record for the *i*-th individual, *μ* is the intercept, *g_i_* is the genotypic effect of the *i*-th individual, and *e* is a random residual .

The genotypic value (*g*) was predicted by regression on the maker types:

where *z_ik_* is the regressor variable for the *i*-th genotype and *k*-th marker, while *u_k_* are the regression coefficients. It was assumed that the regression coefficients are independent random draws from a common normal distribution,

This model was extended to incorporate heterogeneous variances between the *a* (*a* = 5, 10, 50, 100, 250) most significant markers and the remaining *n-a* (*n* = 500, 1000, 2000, 3000) pre-selected markers, similar to model MIXTURE in [5]. The extended model is

where *m*=1 denotes the *a* most significant and *m*=2 the remaining *n-a* pre-selected markers.

The regression coefficients were predicted by best linear unbiased prediction (BLUP) and the variance components estimated by restricted maximum likelihood (REML). For each fitted model we obtained BLUPs for *μ* + *g_i_* corresponding to GEBVs.

### Spatial models

**Figure 1:**
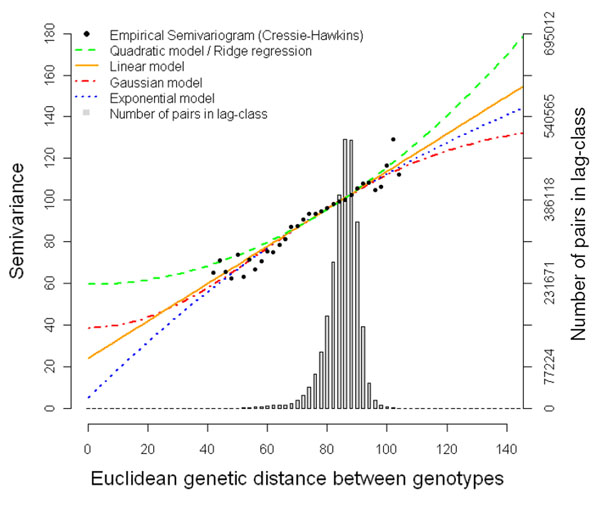
**Empirical semivariogram of the QTL-MAS 2010 dataset and theoretical models (Quadratic, Linear, Gaussian and Exponential) fitted by weighted least squares.** Genotypic covariance models of the form **Γ** = {*f*(*d_ii_*_′_)}, where *d* is the Euclidean distance computed from marker data and *θ* is a parameter, are as follows: Quadratic: *f*(*d*) = 1 – *θd*^2^; Linear: *f*(*d*) = 1 – *θd*; Gaussian: *f*(*d*) = exp(-*d*^2^/*θ*^2^); Exponential: *f*(*d*) = exp(-*d*/*θ*).

We considered different models for the variance of ***g***′ = (*g*_1_,*g*_2_,…,*g_G_*), conditionally on the markers ***Z*** = {*z_ik_*}, where *G* is the number of genotypes. All conditional models were of the form  for some matrix **Γ** that is a function of ***Z*** and  is a variance component. The models that were used are identical to those used in [6,7]. The genetic correlation under the spatial models is expressed as **Γ** = {*f*(*d*_*ii*′_)}, where *d*_*ii*′_ is the Euclidean distance of genotypes *i* and *i*′, defined as *d_ii_*_′_ = ||***z_i_*** – ***z_i_*_′_**||, with  equal to the *i*-th row of ***Z***, and *f*(*d*) is some monotonically decreasing function of *d*. Some examples of the function *f*(*d*) are shown in Figure 1 and in [8]. The quadratic model is equivalent to RR [6]. A semivariogram based on genetic Euclidean distances computed from SNP data can be used to inspect the fit of different models for GS. Hence we used the Cressie-Hawkins robust semivariogram estimator [9].

### Cross-validation

A 5-fold cross-validation (CV) was performed to evaluate model performance. All phenotyped individuals were included in the CV, except those in the first generation. Overall 75 crosses (full sib families) were included. The dataset was randomly split into 5 subsamples each of which contained 15 crosses. In each CV round the phenotypic records for one of the five subsamples was held out and used as a validation set. Each subsample was held out and used as a validation set only once.

The mean Pearson correlations between the GEBVs and observed values in the 5 replicates of the validation sets and between the true breeding values (TBVs) of the non-phenotyped individuals of the fifth generation and GEBVs were used as measures of accuracy.

All mixed models were fitted using the REML method in the SAS MIXED procedure and the theoretical semivariograms in the SAS NLIN procedure.

## Results

A high correlation was established between the semivariance and the genetic distance between pairs of individuals (Fig. 1), suggesting that it is reasonable to model the genetic covariance between pairs of individuals. However, RR and the spatial models gave essentially similar fits (Table 1).

**Table 1:** Selection of different genetic covariance models using Pearson correlations between GEBVs and observed values in the validation sets (CV), and between GEBVs and TBVs for non-phenotyped individuals (TBV). Considered were either all (*n* = 9570) or subsets (*n* = 500, 1000, 2000, 3000) of the 9570 markers, selected by method 2.

	Ridge Regression		Gaussian		Exponential		Linear	
n	CV	TBV	CV	TBV	CV	TBV	CV	TBV

9570	0.530	0.607	0.530	0.600	0.530	0.607	Did not converge	
500	0.570	0.599	0.569	0.596	0.572	0.599	0.572	0.596
1000	0.583	0.623	0.583	0.614	0.583	0.620	0.584	0.614
2000	0.579	0.625	0.580	0.614	0.582	0.621	0.582	0.614
3000	0.576	0.617	0.577	0.608	0.580	0.615	0.580	0.608

**Figure 2:**
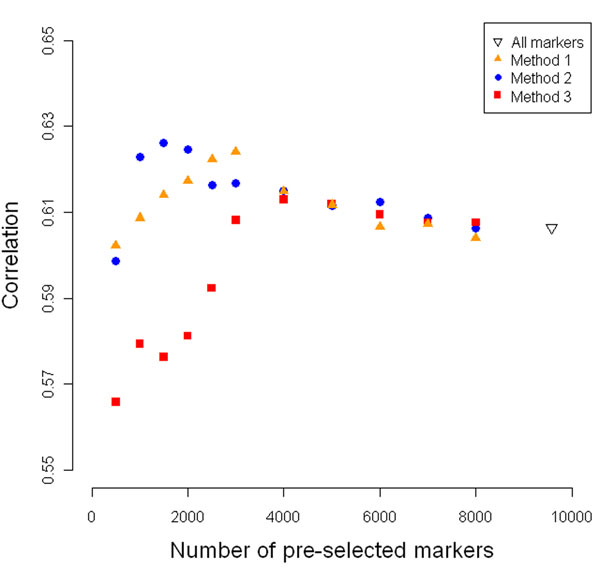
**Mean Pearson correlation between GEBVs and TBVs for non-phenotyped individuals**. GEBVs were estimated by ridge regression.

Pre-selection of markers was evidently beneficial, with methods 1 and 2 achieving similar predictive accuracies and outperforming method 3 (Fig. 2). Method 2 was somewhat better supported than method 1. Comparisons of the GEBVs to the TBVs suggested that it was preferable to pre-select 1000 or 2000 markers for all models, confirming the results of the CV (Table 1).

Moreover, the extended model with heterogeneous variances between lowly and highly significant markers increased accuracy (Table 2).

**Table 2:** Selection of different combinations of pre-selected markers by method 2 (*n* = 1000 or 2000), each partitioned into two groups with different variances, namely *a* (*a* = 0,5,10,50,100, 250) most significant markers and *n-a* markers. Only RR was used to estimate GEBVs. The selection criteria are the same as for Table 1.

Combination		Pearson correlation	
n	a	CV	TBV
1000	0	0.583	0.623
1000	5	0.582	0.625
1000	10	0.586	0.632
1000	50	0.587	0.635
1000	100	0.586	0.637
1000	250	0.584	0.630
2000	0	0.579	0.625
2000	5	0.580	0.628
2000	10	0.588	0.640
2000	50	0.589	0.645
2000	100	0.590	0.648
2000	250	0.588	0.640

Overall, RR with 2000 markers selected by method 2 and allowing for heterogeneous variances among the 100 most significant and the remaining 1900 markers gave the most accurate prediction of GEBVs for the fifth generation.

## Discussion

We have evaluated how pre-selection of markers influences predictive accuracy in GS using RR and its spatial extensions via genetic distances. The spatial models differed in terms of the theoretical models used to model the empirical semivariogram among the genotypes as a function of their genetic distances of separation. All the fitted theoretical semivariogram models were remarkably similar within the range of the observed semivariogram values, and so were their predictions. This suggests that further study is needed to decide if modelling genetic covariances using non-linear spatial models is beneficial compared to RR, especially for non-additive genetic effects.

Our results reinforce findings of other studies suggesting that pre-selecting markers may enhance predictive accuracy [3]. For example, the results of a BLUP model [4] using pre-selected markers were better supported than those of BLUP methods that used all markers [10]. However, pre-selecting markers may not always increase accuracy and may sometimes even reduce it [11].

The extended model with two variance components for the markers increased predictive accuracy because it better approximated the simulated genetic model with a few QTLs with different variances. Heterogeneous variance models may, however, not always exhibit superior performance. In particular, simulating many QTLs with small effects may lower the performance of models allowing for heterogeneous variances among individual markers [5].

## Conclusions

Pre-selection of markers was beneficial and increased predictive accuracy from 0.607 to 0.625. Partitioning markers into two groups with heterogeneous variances further increased accuracy up to 0.648 for the simulated dataset.

## Competing interests

The authors declare no competing interests.

## Authors' contributions

TSS participated in the design of the study, performed all analyses and drafted the manuscript. JOO helped draft the manuscript and interpret the results. HPP conceived the study, participated in its design, and helped in the final editing of the manuscript.
